# Olives and Olive Oil Are Sources of Electrophilic Fatty Acid Nitroalkenes

**DOI:** 10.1371/journal.pone.0084884

**Published:** 2014-01-14

**Authors:** Marco Fazzari, Andrés Trostchansky, Francisco J. Schopfer, Sonia R. Salvatore, Beatriz Sánchez-Calvo, Dario Vitturi, Raquel Valderrama, Juan B. Barroso, Rafael Radi, Bruce A. Freeman, Homero Rubbo

**Affiliations:** 1 Department of Pharmacology and Chemical Biology, University of Pittsburgh School of Medicine, Pittsburgh, Pennsylvania, United States of America; 2 Fondazione Ri.MED, Palermo, Italy; 3 Departamento de Biología Experimental, Universidad de Jaén, Jaén, Andalucía, Spain; 4 Departamento de Bioquímica and Center for Free Radical and Biomedical Research, Facultad de Medicina, Universidad de la República, Montevideo, Uruguay; University of California Riverside, United States of America

## Abstract

Extra virgin olive oil (EVOO) and olives, key sources of unsaturated fatty acids in the Mediterranean diet, provide health benefits to humans. Nitric oxide (•NO) and nitrite (NO_2_^−^)-dependent reactions of unsaturated fatty acids yield electrophilic nitroalkene derivatives (NO_2_-FA) that manifest salutary pleiotropic cell signaling responses in mammals. Herein, the endogenous presence of NO_2_-FA in both EVOO and fresh olives was demonstrated by mass spectrometry. The electrophilic nature of these species was affirmed by the detection of significant levels of protein cysteine adducts of nitro-oleic acid (NO_2_-OA-cysteine) in fresh olives, especially in the peel. Further nitration of EVOO by NO_2_^−^ under acidic gastric digestive conditions revealed that human consumption of olive lipids will produce additional nitro-conjugated linoleic acid (NO_2_-cLA) and nitro-oleic acid (NO_2_-OA). The presence of free and protein-adducted NO_2_-FA in both mammalian and plant lipids further affirm a role for these species as signaling mediators. Since NO_2_-FA instigate adaptive anti-inflammatory gene expression and metabolic responses, these redox-derived metabolites may contribute to the cardiovascular benefits associated with the Mediterranean diet.

## Introduction

Olive oil is the principal source of lipids in the Mediterranean diet, with “extra virgin” olive oil (EVOO) referring to an oil fraction produced via mechanical rather than chemical extraction of olives at temperatures that limit effects on intrinsic properties of the oil [1], [2]. The principal components of EVOO are triglycerides (TG, 98–99%) predominantly esterified with monounsaturated oleic acid (OA), and to a lesser extent palmitic, linoleic (LA) and linolenic acids [3]–[5].

Multiple health benefits are linked with diets rich in olive oil and the Mediterranean diet in general, including anti-inflammatory and anti-hypertensive effects that lead to a reduced risk of cardiovascular morbidity and mortality [6]–[8]. Notably, the Mediterranean diet is also linked with the consumption of fruits and vegetables that are rich in the inorganic anions nitrite (NO_2_^−^) and nitrate (NO_3_^−^) [9], [10]. These species are also metastable nitric oxide (NO) oxidation products in vivo. Collectively, these oxides of nitrogen undergo further reactions in the blood and tissues via enzymatic and non-enzymatic reductive metabolism and by the oxidizing, nitrosating and nitrating conditions promoted by digestion, mitochondrial respiration and inflammation [11]. In the case of NO_3_^−^, the commensal bacteria of the enterosalivary system reduce dietary NO_3_^−^ to physiologically-significant levels of NO_2_^−^, NO and secondary species [12]. There is also an emerging body of evidence from higher plants that NO and other reactive species mediate nitro-oxidative reactions that regulate plant stress perception, signal transduction and senescence responses. Integral to these events is the redox-mediated formation of heme and protein thiol nitrosyl adducts and protein 3-nitrotyrosine adducts [13]. Considering that plants in general, and fresh olives in particular, are abundant in readily-nitrated unsaturated fatty acids, the present study evaluated whether electrophilic fatty acid nitroalkene derivatives (NO_2_-FA) are a) endogenously present in olives, b) extractable into the EVOO fraction and c) generated after consumption of olive lipids by the acidic conditions of digestion.

Fatty acid nitroalkenes are detectable clinically and in rodent models as free, esterified and protein-adducted species [14], but have not been reported in plants. In mammals, these species are present in low basal concentrations and are formed at greater concentrations by the radical addition reaction of nitrogen dioxide (NO_2_) to unsaturated fatty acids. The endogenous generation of NO_2_ occurs via multiple acid-catalyzed and oxidative inflammatory reactions involving NO and NO_2_^−^ [15]. Biochemical studies and cell models revealed that NO_2_-FA are electrophilic, if the nitro group is adducted to alkenyl carbons. These species rapidly react via Michael addition with thiols and to a lesser extent primary and secondary amines [16].

Once generated, NO_2_-FA signal by reversibly alkylating susceptible thiols of multiple transcriptional regulatory proteins, thus affecting downstream gene expression and the metabolic and inflammatory responses under their regulation. Via this mechanism, NO_2_-FA activate Nrf2-dependent antioxidant gene expression by adduction of critical thiols in the Nrf2 regulatory protein Keap1 [17]. NO_2_-FA also inhibit pro-inflammatory cytokine, adhesion protein and enzyme expression by adduction of the NF-κB p65 subunit and inhibition of DNA binding by p65 [18]. NO_2_-FA are also partial agonists of peroxisome proliferator-activated receptor-γ (PPARγ), which NO_2_-FA activate via hydrogen bonding interactions and covalent adduction of the ligand binding domain Cys285 [19]. Finally, NO_2_-FA limit inflammatory responses by non-cGMP-dependent inhibition of platelet and neutrophil function [20], [21] and by inhibiting the catalytic activity and gene expression of the pro-inflammatory proteins cyclooxygenase-2 and xanthine oxidoreductase [22], [23]. Via these pleiotropic mechanisms, murine models reveal that NO_2_-FA limit pathologies linked with obesity, ischemic episodes, bacterial lipopolysaccharide and surgical procedures such as angioplasty [24]–[29].

Herein, we report the endogenous presence of NO_2_-FA in plants, specifically in olives and EVOO and show the additional formation of NO_2_-FA from EVOO under conditions which mimic gastric pH and NO_2_^−^ concentrations during digestion. It is speculated that the dietary consumption and physiologic generation of electrophilic anti-inflammatory lipids contribute to the physiological benefits of unsaturated fatty acid-rich diets.

## Materials and Methods

### Materials

All chemicals were purchased from Sigma-Aldrich (St. Louis, MO, USA) if not stated otherwise. All nitro fatty acids and internal standards NO_2_-[^13^C_18_]OA, NO_2_-[^13^C_18_]LA and [^15^N]O_2_-cLA were synthesized as previously [30]–[32]. Pancreatic lipase, cysteine, [^13^C_3_,^15^N]cysteine and methanesulphonic acid were purchased from Sigma-Aldrich (L3126, C122009, 658057, 471356). Gastric juice artificial was purchased from Fisher Scientific Company (S76772). Strata NH_2_ (55 µm, 70A) columns were from Phenomenex (8B-S009-HCH). Hypersep C18 columns were purchased from Thermo Scientific (60108-305). Mass spectrometry quality solvents were purchased from Burdick and Jackson (Muskegon, MI, USA). Extra virgin olive oils and fresh olives were from Jaen, Spain, and came from three different types of cultivars: Arbequina, Frantoio and Picual [33].

### Storage of EVOO

To assure no further oxidation of EVOO occurred during storage, α-tocopherol (α-TOH) was determined in fresh samples stored in the dark at either −20°C or room temperature. The levels of α-TOH were determined by reverse phase HPLC of samples (50 μl) mixed with methanol (450 μl) and vortexed twice for 10 s, centrifuged at 10,000 x g for 10 min at 4°C. α-TOH was resolved on a Supelcosil LC-18 column (25×0.46 cm, 5 µm), mobile phase of 100% methanol at a flow rate of 1 ml/min. Fluorescence detection (λ_exc_ = 295 nm, λ_em_ = 330 nm), comparing peak areas with corresponding standards [34]. α-TOH levels were stable for at least two weeks at -20°C in contrast to storage at room temperature.

### In vitro gastric digestion of EVOO

Olive oil (10 µl) was incubated for 1 h at 37°C in 1 ml of gastric juice artificial with 5 mM Na[^15^N]O_2_, under continuous agitation [32], [35]. The lipid fraction was extracted by hexane, dried under a stream of nitrogen and 1 ml pancreatic lipase (0.4 mg protein/ml) in 0.5 M phosphate buffer, pH 7.4 was added and the reaction mixture incubated at 37°C for 3 h, under agitation. The lipid fraction was extracted by Bligh and Dyer method [36], dried under a stream of nitrogen and dissolved in chloroform. Lipid classes were further resolved by solid phase extraction (SPE) Strata NH_2_ columns. Briefly, columns were pre-conditioned with 6 ml hexane, followed by 6 ml chloroform/isopropanol (2∶1, v/v); samples were added and the column was washed with other 6 ml chloroform/isopropanol (2∶1, v/v). Then, free fatty acids were eluted with 6 ml diethylether/2% acetic acid. The solvent was evaporated under a stream of nitrogen and lipids were dissolved in methanol for HPLC-ESI-MS/MS and high resolution mass spectrometry analysis.

### Detection and characterization of fatty acid nitroalkenes in EVOO

Analysis of NO_2_-FA was performed by HPLC-ESI-MS/MS using a triple quadrupole mass spectrometer (API4000, Applied Biosystems, Framingham, MA) in parallel with a LTQ Orbitrap Velos (Thermo Scientific) in negative ion mode. NO_2_-FA in lipid extracts were separated using a C18 reverse phase column (2×150 mm, 3 µm, Phenomenex) eluted at a flow rate of 0.25 ml/min using a solvent system consisting of A (H_2_O/0.1% acetic acid) and B (acetonitrile/0.1% acetic acid), with the following solvent gradient: 45% B (0–0.1 min); 45–80% B (0.1–45 min); 80–100% B (45–46 min); 100% B (46–47 min) and then columns were re-equilibrated to initial conditions for an additional 10 min. The triple quadrupole mass spectrometer was set with the following parameters: declustering potential (DP) of –65 V, collision energy (CE) of –35 eV and a desolvation temperature of 650°C. Detection of NO_2_-FA was performed via MRM scan mode with specific MRM transitions corresponding to the potential nitrated isomers of OA, LA and cLA [30], [32]. In all cases, data was acquired, analyzed and processed using Analyst 1.5.1 software (Applied Biosystems, Framingham, MA) as previously [32], [35]. High resolution mass spectrometry analysis was performed using the LTQ Orbitrap Velos equipped with a HESI II electrospray source. The following parameters were used: heater temperature 200°C, capillary temperature 200°C, sheath gas flow rate 6, auxiliary gas flow rate 10, sweep gas flow rate 5, source voltage −6 kV, S-lens RF level 65%. The instrument calibration in FT-mode was performed with manufacture calibration solutions. Data were acquired, analyzed and processed using Xcalibur 2.1 software (Thermo Scientific) as previously [37].

### Analysis of conjugated linoleic acid in EVOO

cLA was detected by the formation of Diels-Alder adducts with PTAD [32]. EVOO samples were incubated in chloroform with PTAD for 2 min at room temperature and reactions were stopped by the addition of 1,3-hexadiene [32]. Solvent was evaporated and samples washed three times with methanol followed by digestion with pancreatic lipase and SPE as before. Samples were diluted in methanol and analyzed by HPLC-MS/MS by following the specific MRM transition for different PTAD-derivatized cLA isomers [32].

### Nitro-fatty acid-cysteine reaction analysis

NO_2_-OA (100 µM) and [^15^N]O_2_-cLA (100 µM) reaction with 25 mM cysteine was conducted in 50 mM phosphate buffer, pH 7.4 for 1 h at 37°C. The same reaction was conducted using NO_2_-OA and [^13^C_3_,^15^N]cysteine. Reactions were acidified with formic acid to pH 3 to stop the Michael additions and NO_2_-FA cysteine adducts detected by HPLC-MS/MS following the specific MRM transitions: 447/120 and 451/124, corresponding to NO_2_-OA-cysteine and its internal standard NO_2_-OA-[^13^C_3_,^15^N]cysteine. For fresh olive analysis, whole olives, mesocarp and peel were obtained and homogenized in 50 mM phosphate buffer, pH 7.4. Protein fraction was extracted three times by Bligh and Dyer method [36], then 1 ml hexane was added and proteins were sedimented by centrifugation at 1890 x g for 10 min, washed with 2 ml methanol/water (4∶1, v/v), centrifuged and resuspended in 1 ml water. An equivalent volume of 8 M methanesulphonic acid and internal standard was added and the samples were hydrolyzed for 6 h at 110°C. After hydrolysis, the samples were diluted to 15% methanol and NO_2_-FA-cysteine adducts purified by SPE using Hypersep C18 columns. Columns were pre-conditioned with 6 ml methanol, followed by 6 ml 15% methanol in 1% formic acid; then samples were loaded, washed with 6 ml 15% methanol/1% formic acid and columns were dried under vacuum for 30 minutes. NO_2_-FA-cysteine adducts were then eluted with 3 ml methanol in 1% formic acid and the solvent was evaporated at room temperature under a stream of nitrogen. Samples were dissolved in 100 µl methanol and analyzed by HPLC-MS/MS using a triple quadrupole mass spectrometer (API4000, Applied Biosystems, Framingham, MA) in negative ion mode with a DP of –100 V, CE of –25 eV and a desolvation temperature of 650°C. NO_2_-FA-cysteine adducts were separated using one of two different elution schemes on a C18 reverse phase column (2×150 mm, 3 µm, Phenomenex). For structural determinations, the mobile phase consisted of solvent A (H_2_O/0.1% acetic acid) and solvent B (acetonitrile/0.1% acetic acid). Chromatography was at 0.25 ml/min with the following solvent gradient: 5% B (0–1 min); 5–35% B (1–8.5 min); 35–100% B (8.51–47 min); 100% B (47–53 min) and re-equilibrated to initial condition for additional 7 min. For quantitative analysis, NO_2_-FA-cysteine adducts were separated using a C18 reverse phase column (2×20 mm, 3 µm, Phenomenex); The same mobile phases were used and chromatography was at 0.75 ml/min using the following solvent gradient: 5% B (0–0.1 min); 5–35% B (0.1–0.85 min); 35–100% B (0.86–4.7 min); 100% B (4.7–5.3 min) and then columns were re-equilibrated to initial conditions for an additional 1.6 min. Detection of NO_2_-FA-cysteine adducts was performed using the MRM scan mode and acquired data analyzed and processed using Analyst 1.5.1 software, as previously [32], [35].

## Results

### Endogenous NO_2_-cLA and cLA in olive oil

**Figure 1:**
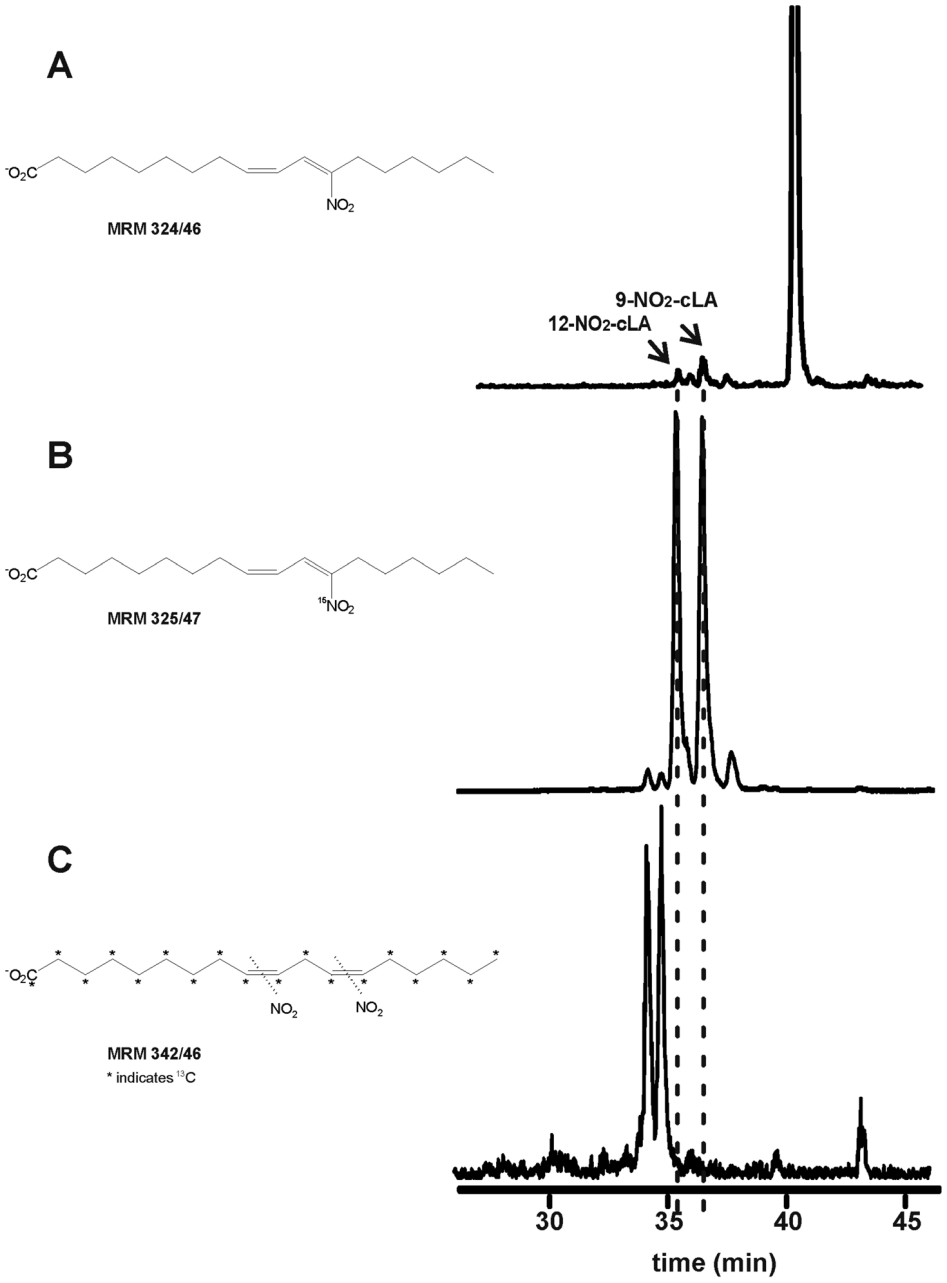
Detection of NO_2_-cLA in EVOO. Extra virgin olive oil was washed four times with methanol and treated with pancreatic lipase for 2-MS/MS. Before extraction, internal standards [^15^N]O_2_-cLA and NO_2_-[^13^C_18_]LA were added. The following MRM transitions were analyzed: 324/46 for NO_2_-LA, 325/47 for [^15^N]O_2_-cLA and 342/46 for NO_2_-[^13^C_18_]LA. The elution profile of (**A**) lipids from EVOO; (**B**) [^15^N]O_2_-cLA and (**C**) NO_2_-[^13^C_18_]LA are shown. Neither NO_2_-LA nor NO_2_-OA (MRM 326/46) was detected. Data is representative of at least 3 independent experiments.

NO_2_-FA content was determined by high performance liquid chromatography- electrospray ionization- tandem mass spectrometry (HPLC-MS/MS) analysis of the free fatty acid fraction of lipase-digested EVOO [33]. Endogenous nitro-conjugated linoleic acid (NO_2_-cLA) was detected by multiple reaction monitoring (MRM) transition 324.2/46 (Figure 1A). These peaks co-eluted with [^15^N]O_2_-cLA (Figure 1B) but not NO_2_-[^13^C_18_]linoleic acid standards (NO_2_-[^13^C_18_]LA, Figure 1C). Analysis by high resolution mass spectrometry and the electrophilic reactivity of this species, determined by thiol reactivity according to [32], [38], further confirmed the endogenous presence of NO_2_-cLA (Figure S1). Nitrite was undetectable in EVOO, purified lipase preparations and all solvents used for extractions and chromatography, discounting the possibility of artifactual fatty acid nitration during sample preparation.

The presence of cLA in EVOO [39], [40] was determined by selective derivatization with 4-phenyl-1,2,4- triazoline-3,5-dione (PTAD) and product analysis by HPLC-MS/MS (Figure S2) (30). The MRM transitions, elution profiles and MS/MS analysis revealed that octadecadi-(*9,11*)-enoic acid (*m/z* 454.4/335.2, *m/z* 454.4/224.2, *m/z* 454.4/191.2 and *m/z* 454.4/168.2) was the predominant cLA regioisomer (Figure S2).

### NO_2_-FA generation from olive oil by modeling digestion ex vivo

**Figure 2:**
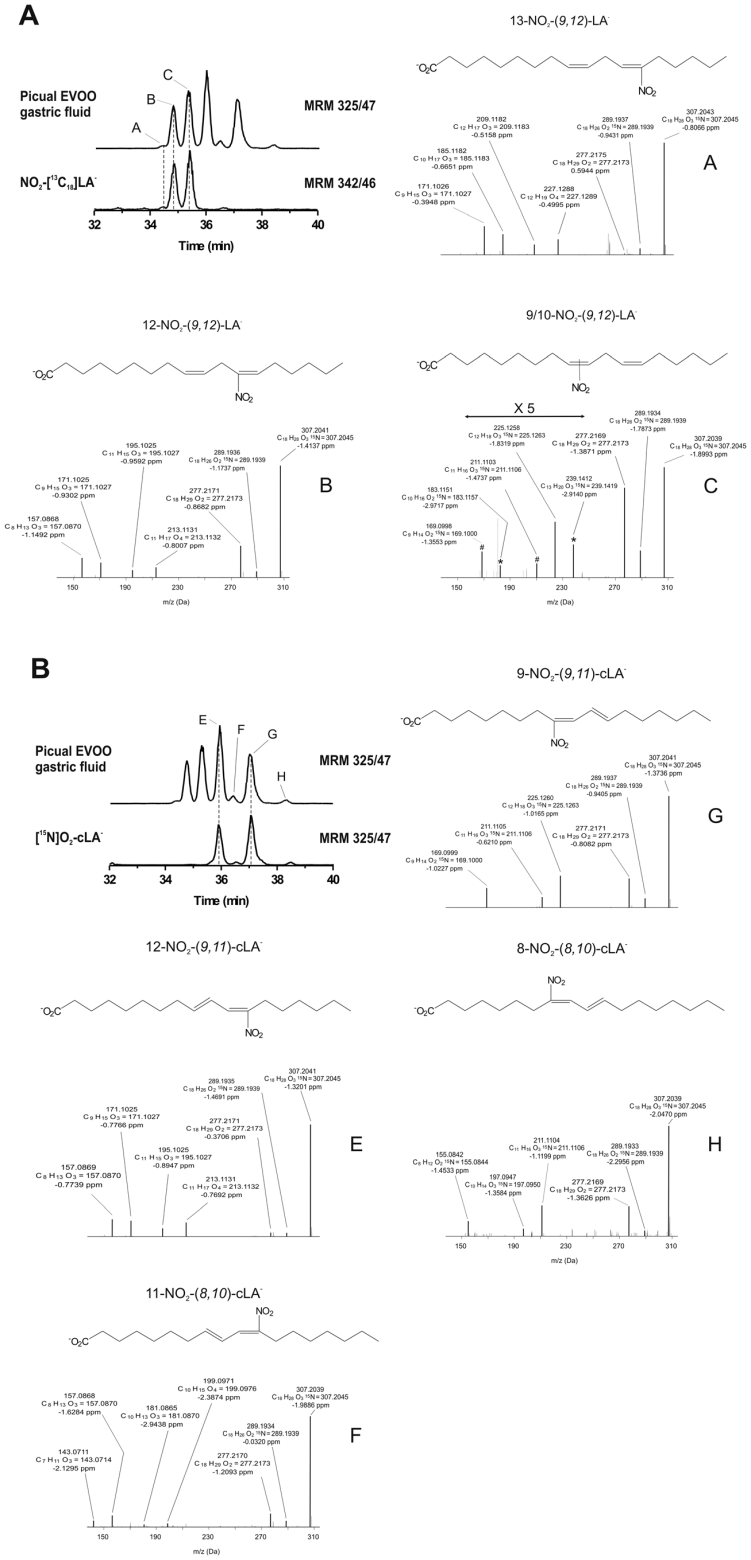
Identification of specific NO_2_-LA and NO_2_-cLA regio-isomers in EVOO by *in vitro* digestion modeling. Olive oil (10 µl) was incubated in gastric juice artificial with 5 mM Na[^15^N]O_2_. The lipid fraction was extracted, dried and incubated with pancreatic lipase (0.4 mg protein/ml) in phosphate buffer, pH 7.4 at 37°C for 3 h. The lipid fraction was extracted, dried, dissolved in chloroform, then solid phase extraction was performed and lipids analyzed by HPLC-MS/MS. The presence of NO_2_-LA and NO_2_-cLA in Picual EVOO gastric fluid was determined following the MRM transition *m/z* 325/47 compared to (A) the internal standard NO_2_-[^13^C_18_]LA (*m/z* 342/46) and (B) the standard [^15^N]O_2_-cLA (*m/z* 325/47). Similar results were obtained for the other two EVOO tested (Arbequina and Frantoio oils). The corresponding peaks were also observed when EVOO was nitrated with NaNO_2_ (data not shown). Data shown are representative of at least 3 independent experiments. (A) Orbitrap Velos analysis confirmed the elemental composition (C_18_H_30_O_4_^15^N) and mass accuracy (−2.0443 ppm) for the peaks labeled A, B, C, and related MS^2^ analysis of each [^15^N]O_2_-LA regio-isomers. # and * represents specific fragments for the 9-NO_2_ and 12-NO_2_ isomers, respectively. (B) For peaks labeled E to H, Orbitrap Velos analysis confirmed the elemental composition (C_18_H_30_O_4_^15^N) and mass accuracy (−2.0923 ppm) and related MS^2^ analysis of [^15^N]O_2_-cLA regio-isomers.

**Figure 3:**
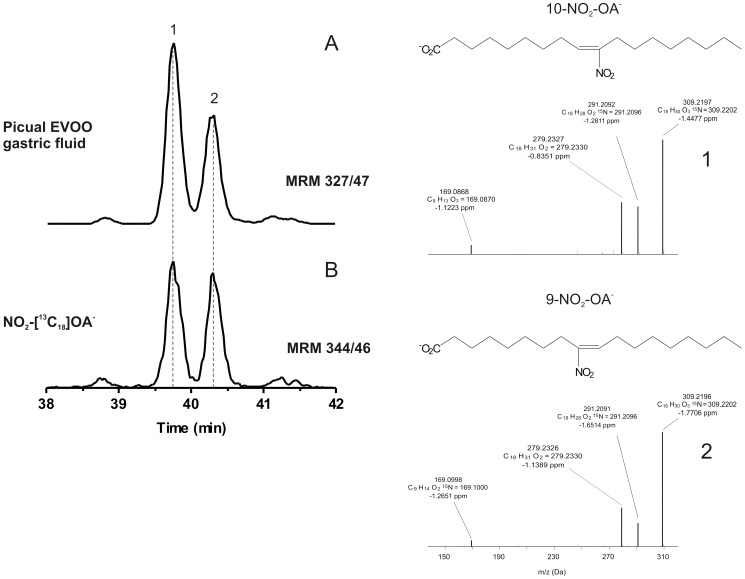
NO_2_-OA generation from EVOO by *in vitro* digestion modeling. Olive oil was nitrated, extracted and treated with Na[^15^N]O_2_ as in Figure 2. (A) The presence of NO_2_-OA in Picual EVOO gastric fluid was determined following the MRM transition *m/z* 327/47 compared to (B) the internal standard NO_2_-[^13^C_18_]OA (*m/z* 344/46). Similar results were obtained for the other EVOOs tested. The corresponding peaks were also observed when EVOO was nitrated with NaNO_2_. Data shown is representative of at least 3 independent experiments. Orbitrap Velos analysis confirmed the elementalcomposition (C_18_H_32_O_4_^15^N) and mass accuracy (−2.8007 ppm) and related MS^2^ analysis of [^15^N]O_2_-OA regio-isomers.

The acidic milieu of the gastric compartment and the presence of NO_2_^−^ in food promotes a nitrative environment due to HNO_2_ formation (NO_2_^−^ pK_a_, 3.4) that in turn can mediate biomolecule nitration [41], [42]. To model this, EVOO was incubated in gastric juice with [^15^N]O_2_^−^ and the generation of NO_2_-FA was determined (Figures 2 and 3). Under these conditions, seven principal NO_2_-FA ions were detected by following the MRM transition 325.2/47 (Figure 2). The first three peaks displayed the same retention time as the internal standard NO_2_-[^13^C_18_]LA (Figure 2A), while the other peaks coincided with both the synthetic standard NO_2_-cLA (data not shown) and [^15^N]O_2_-cLA (Figure 2B). High resolution MS analysis at the 2 ppm level confirmed the elemental composition of [^15^N]O_2_-LA and [^15^N]O_2_-cLA while MS^2^ analysis revealed 9-,10-,12-, and 13-NO_2_ positional isomers of [^15^N]O_2_-LA (Figure 2A), 9- and12-NO_2_ regio-isomers of [^15^N]O_2_-octadeca-(9,11)-dienoic acid and traces of 8- and 11-NO_2_ positional isomers of [^15^N]O_2_-octadeca-(8,10)-dienoic acid (Figure 2B). MS^2^ fragmentation of each isomer yielded product ions with loss of H_2_O, 2H_2_O and HNO_2_, specific for NO_2_-FA (Figures 2A and B; ref. [37]). Under these conditions nitro-oleic acid (NO_2_-OA) was also detected (Figure 3). Specifically, [^15^N]O_2_-OA formation was revealed by the MRM transition 327.2/47. Two peaks (39.7, 40.3 min) shared the same retention time as the standard NO_2_-[^13^C_18_]OA (Figure 3). High resolution MS analysis at the 3 ppm level confirmed the elemental composition of [^15^N]O_2_-OA and MS^2^ analysis showed the presence of both 9- and 10-NO_2_ regio-isomers of NO_2_-OA (Figure 3; ref. [37]).

### NO_2_-FA-cysteine adducts in proteins of fresh olives

**Figure 4:**
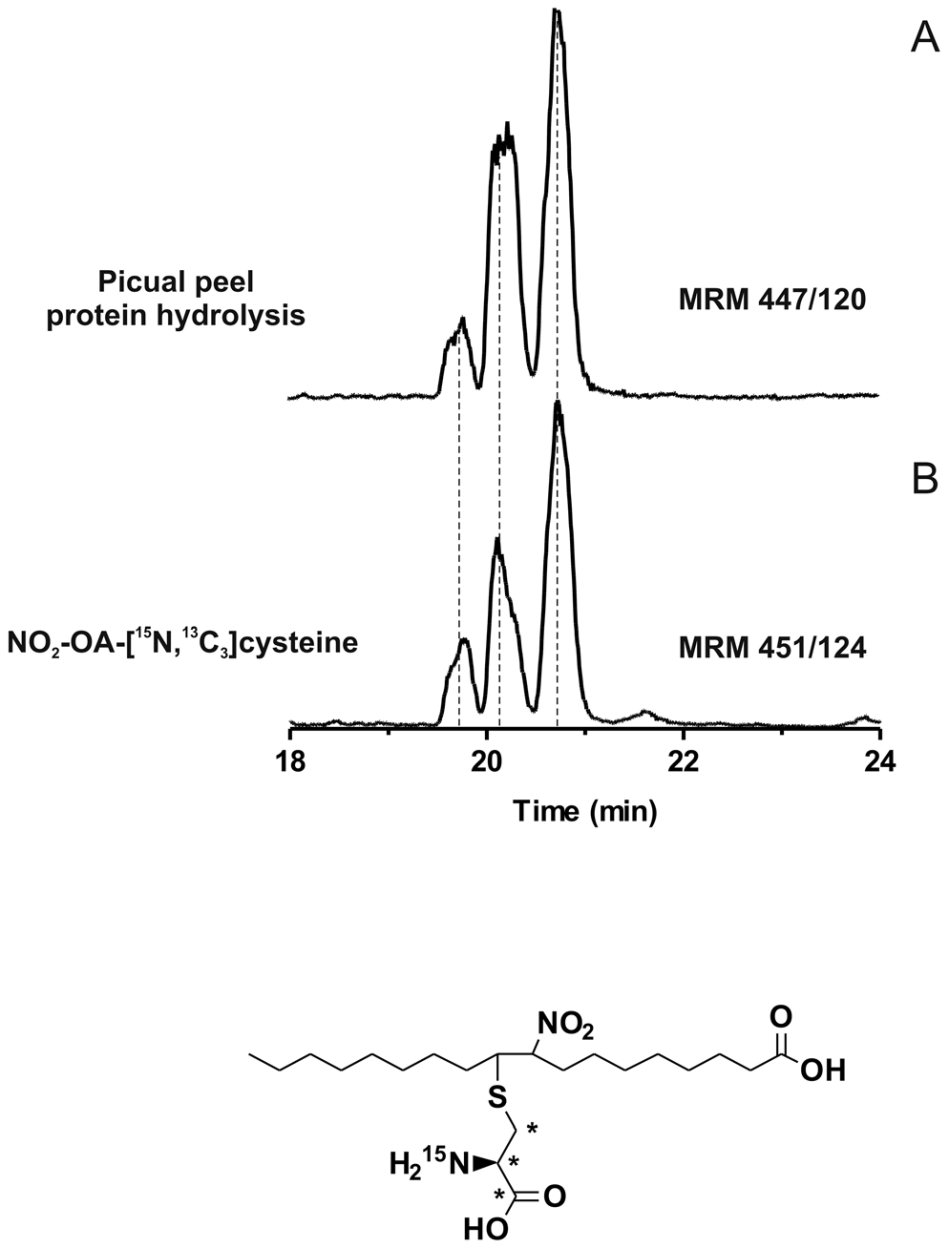
Detection of NO_2_-OA-cysteine adducts in fresh olives. Whole olives, mesocarp and peel were homogenized and protein fraction was extracted and hydrolyzed with methanesulphonic acid for 6°C. Then, solid phase extraction was performed and NO_2_-OA-cysteine adducts were analyzed by HPLC-MS/MS. (A) The presence of NO_2_-OA-cysteine adducts in Picual peel after protein hydrolysis was determined following the MRM transition *m/z* 447/120 compared to (B) the internal standard NO_2_-OA-[^13^C_3_,^15^N]cysteine adducts (*m/z* 451/124). Data shown is representative of at least 3 independent experiments.

**Figure 5:**
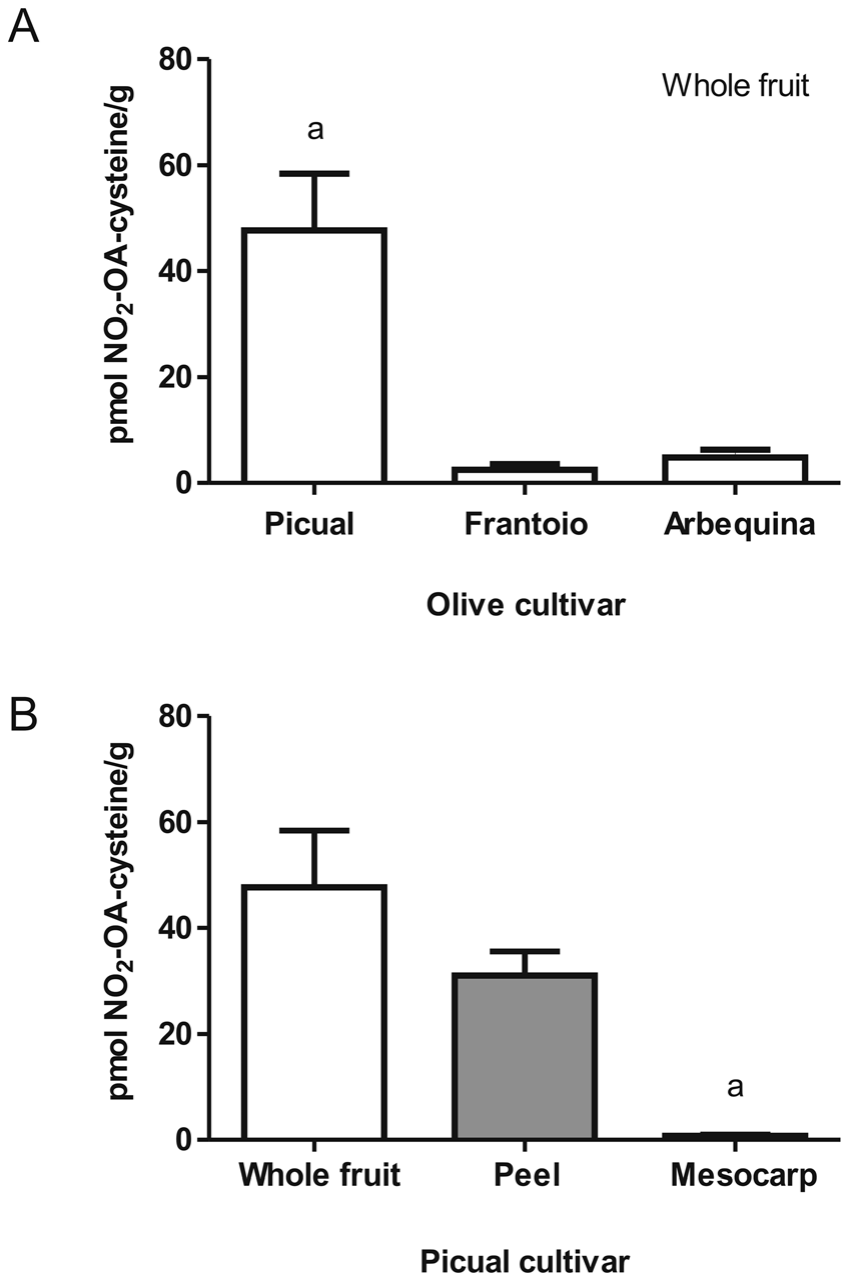
Endogenous NO_2_-OA-cysteine adducts in fresh olives. Whole olive as well as peel and mesocarp fractions were processed as before, and the presence of the NO_2_-OA-cysteine adducts were quantitated by HPLC-MS/MS. (A) NO_2_-OA-cysteine adducts were quantitated in the whole fruit of the three olive cultivars tested. (B) Distribution of NO_2_-OA-cysteine adducts in whole fruit, peel and mesocarp of Picual olive cultivar is shown. Data plotted correspond to the mean ± SD (n = 3) and is representative of at least 3 independent experiments. Statistical analysis was performed by one-way ANOVA with Bonferroni post hoc comparisons. a, p <0.01.

MS analysis of acid-hydrolyzed proteins from freshly-picked whole olive, peel and mesocarp showed three chromatographic peaks having a MRM transition of 447/120, specific for NO_2_-OA-cysteine adducts (Figure 4). These products co-eluted with the synthetic internal standard NO_2_-OA-[^13^C_3_,^15^N]cysteine. NO_2_-OA-cysteine levels varied between different cultivars (Figure 5A). The peel had the greatest NO_2_-OA-cysteine content (up to ∼50 pmol NO_2_-OA-cysteine/g) (Figure 5B). Artifactual nitration reactions induced by the hydrolysis procedure was controlled for by adding 1 μM Na[^15^N]O_2_ and monitoring the potential formation of [^15^N]O_2_-OA- cysteine, which was undetectable.

## Discussion

Olive oil, the principal fat in the Mediterranean diet, promotes anti-inflammatory responses and clinical benefit via poorly-defined mechanisms [1], [43]. This study examined the endogenous content of NO_2_-FA in fresh olives and EVOO and the potential for their further formation when EVOO was subjected to the gastric milieu [24], [26], [30], [44]–[50]. These analyses were motivated by the detection of fatty acid nitroalkenes in rodents and humans and an appreciation that these electrophilic species induce beneficial metabolic and inflammatory signaling responses.

To avoid ion suppression from native fatty acids in the HPLC-MS/MS identification of the less abundant fatty acid nitroalkenes in EVOO, glycerides were hydrolyzed by triacylglycerol lipase to free fatty acids that were then subjected to chromatographic separation. While monoenoic OA is the most abundant fatty acid in EVOO, it is less susceptible to nitration than polyenoic fatty acids. Free and esterified NO_2_-OA was not detected under basal conditions in the EVOO from three different cultivars. Two predominant nitro-containing fatty acid ions with the MRM transition 324/46 were detected. These species displayed retention times and fragmentation characteristics corresponding to the [^15^N]O_2_-cLA standard (Figure 1A,B) and not the bis-allylic NO_2_-[^13^C_18_]LA standard (Figure 1C). cLA consists of positional isomers of linoleic acid having conjugated dienes in the *cis* and *trans* configurations [39], [51], [52]. There are both plant and animal-derived sources of cLA in the human diet. In addition, both mammalian and enterosalivary microbiome enzymes can synthesize conjugated diene fatty acids in humans both de novo and by desaturation of monoenoic fatty acid substrates [39], [40]. Plant lipids have cLA levels of up to ∼1.0 mg cLA/g fat [39], with reported levels of cLA in olive oils of up to 0.2 mg/g fat [39], predominantly the *cis*9-, *trans*11- and *trans*10-, *cis*12- isomers [39]. The presence of cLA in the oils from the three olive cultivars studied herein was confirmed by HPLC-MS/MS detection of a Diels Alder reaction of conjugated dienes with 4-phenyl-1,2,4-triazoline-3,5-dione (PTAD, *m/z* 454) (Figure S2 [32]).

In addition to olives and olive oil, the Mediterranean diet is also rich in NO_2_^-^ and NO_3_^−^-containing vegetables, suggesting that NO_2_-FA generation could also occur by acidic nitration reactions in the stomach [1], [41], [43]. NMR analysis of EVOO has suggested that nitro-oxidative modifications of the phenolic and glyceride constituents could yield nitroalkene, nitroalkane and nitro-hydroxy products [53]. Herein, the formation of electrophilic NO_2_-FA species in EVOO exposed to Na[^15^N]O_2_ in gastric fluid was detected with HPLC-MS/MS (Figures 2 and 3). Under these conditions, there was significant NO_2_-OA, NO_2_-LA and NO_2_-cLA generation, with 9-NO_2_-cLA and 12-NO_2_-cLA the most prevalent (Figure 2B).

The electrophilic character of NO_2_-FA in EVOO was confirmed by HPLC-MS/MS detection of Michael reaction products in fresh olives (Figure 4) [38]. Notably, NO_2_-OA-cysteine adducts were endogenously present in olives, where there was a stable pool of protein-adducted nitroalkene derivatives in the peel and mesocarp of different olive cultivars (Figure 5). These results confirm that EVOO and olives are both a source and metabolic reserve of NO_2_-FA.

Electrophilic nitroalkenes exert signaling actions via the modulation of the expression and activity of both anti-inflammatory [32] and pro-inflammatory enzymes [23]. These effects are entirely dependent on the post-translational modification of transcription factors, enzymes and and other protein targets via Michael addition. Conjugated linoleic acid displays immune-modulatory and anti-inflammatory effects [51], [52], with the mechanisms accounting for these actions proposed to include the reduction of pro-inflammatory cytokine levels via inhibition of NF-κB-dependent gene expression and activation of PPAR-regulated gene expression [51], [52]. Of significance, very high and non-physiological concentrations of native cLA and other unsaturated fatty acids are required to exert these effects. In contrast, after fatty acid nitration and conferral of electrophilic reactivity, NO_2_-FA potently modulate these same pathways at nM concentrations [46], [54]. Additionally, NO_2_-FA activate Nrf2-regulated anti-inflammatory gene expression and heat shock factor-regulated heat shock protein expression [17], [46], [55]. This is explained by the facile and reversible Michael addition of NO_2_-FA with susceptible thiols of specific protein targets, thus requiring only low concentrations and rates of generation of electrophilic lipids to result in the accumulation of target protein adduction and instigation of downstream signaling responses [16], [54]. Consequently, electrophilic lipids which are present in plants, generated by digestion of plant lipids, produced by oxidative inflammatory reactions or administered as pure synthetic homologs [24], [26], [47], [56], can regulate metabolism and the resolution of inflammatory processes. In this regard, olives and EVOO serve as both a direct source of and precursors for NO_2_-FA generation.

## Supporting Information

Figure S1 **High resolution mass spectrometry analysis of NO_2_-cLA in EVOO.** Extra virgin olive oil was hydrolyzed and extracted by solid phase extraction for HPLC-MS/MS analysis. (A) The presence of NO_2_-cLA in EVOO was confirmed by comparing to the internal standards NO_2_-[^13^C_18_]LA and [^15^N]O_2_-cLA. (B) FTMS-pESI full MS analysis exhibited the presence of a product with the expected mass and composition for NO_2_-cLA as shown in the spectra.
(TIF)

Figure S2 **Conjugated linoleic acid is present in EVOO.** Arbequina oil was incubated with 0.1 mM PTAD for 10 min and the reaction stopped by the addition of 0,2 mM 1,3-hexadiene. Lipids were extracted, treated with pancreatic lipase as previously and analyzed by MS/MS. The presence of conj-LA was followed by the specific MRM transition m/z 454/335; the identification of the specific isomer (A) c9-, t11-conj-LA ((9*Z*, 11*E*)-cLA) was monitored at m/z 454/191, 454/224 and 454/168. (B) These results were confirmed by performing enhanced product ion analysis of the eluted peak, The specific fragments of *m/z* 454, *m/z* 335, *m/z* 224, *m/z* 206, *m/z* 191 and *m/z* 168 confirmed that EVOO contains (9*Z*, 11*E*)-cLA (37). Similar results were obtained for the other two EVOO tested. Data shown is representative of at least 3 independent experiments. (C) corresponds to an scheme of the reaction of conjugated dienes with PTAD.
(TIF)
