# Supplementary figures and images for: Olives and Olive Oil Are Sources of Electrophilic Fatty Acid Nitroalkenes
Source: PLoS One. 2014 Jan 14;9(1):e84884. doi: 10.1371/journal.pone.0084884 (PMC3891761; doi:10.1371/journal.pone.0084884)

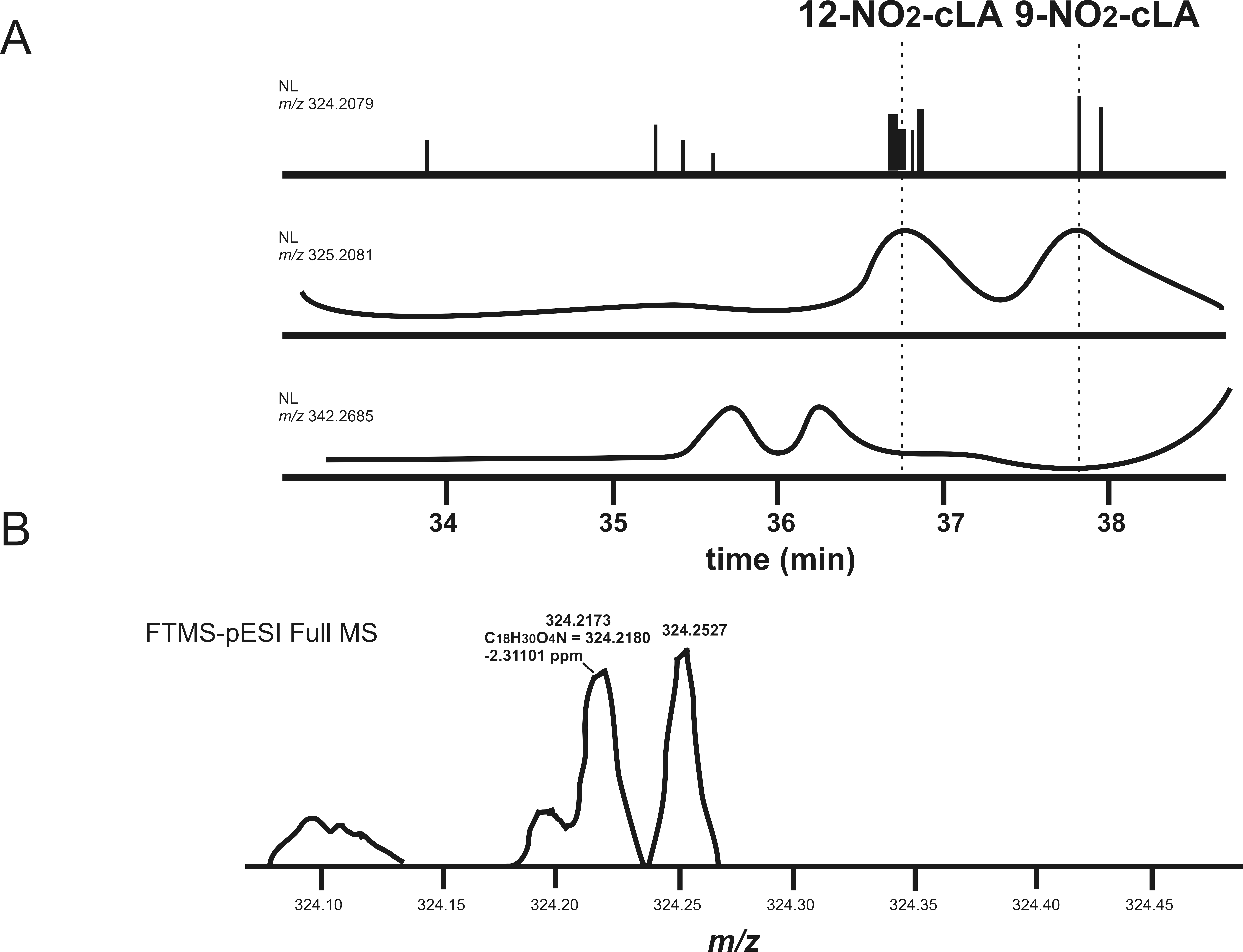

Supplement: Figure S1 — High resolution mass spectrometry analysis of NO2-cLA in EVOO. Extra virgin olive oil was hydrolyzed and extracted by solid phase extraction for HPLC-MS/MS analysis. (A) The presence of NO2-cLA in EVOO was confirmed by comparing to the internal standards NO2-[13C18]LA and [15N]O2-cLA. (B) FTMS-pESI full MS analysis exhibited the presence of a product with the expected mass and composition for NO2-cLA as shown in the spectra. (TIF) [file pone.0084884.s001.tif]
